# The Common Neurological Presentations and Clinical Outcomes of Coronavirus Disease 2019 in Saudi Arabia

**DOI:** 10.3389/fneur.2021.737328

**Published:** 2021-09-08

**Authors:** Walid A. Alkeridy, Mohammed H. Alanazy, Nada Alamri, Awyshah Alqahtani, Adel Alhazzani, Taim Muayqil

**Affiliations:** ^1^Department of Medicine, King Saud University, Riyadh, Saudi Arabia; ^2^Department of Medicine, Geriatric Division, University of British Columbia, Vancouver, BC, Canada

**Keywords:** COVID-19, delirium, encephalopathy, Saudi Arabia, neurological

## Abstract

**Background:** Neurological manifestations have increasingly become recognized in COVID-19. People from different ethnic backgrounds are experiencing different outcomes related to SARS-CoV-2 infection. Several cohort studies reported the common neurological manifestations and complications associated with COVID-19 disease around the world however, the prevalence of neurological complications associated with SARS-CoV-2 infection in the Arab countries and Saudi Arabia is still unknown.

**Objective:** To study the prevalence, risk factors, and characteristics of the neurological complications associated with COVID-19 and their relationship with clinical outcomes.

**Methods:** We conducted a prospective, single-center, observational, cohort study of consecutive hospitalized adults COVID-19 patients with and without neurological manifestation admitted between March 2020 until the end of December 2020. Data was collected prospectively using electronic medical records; Cases and controls were observed until they either get discharged from the hospital or died. The primary outcomes were death, survival, and survival with sequalae.

**Results:** Among 497 patients with COVID-19, 118 patients (23.7%) had neurological complications, 94 patients (18.9%) had encephalopathy, and 16 patients (3.2%) had cerebrovascular accidents (CVA). Patients with COVID-19-related neurological complications were older and more likely to have a pre-existing neurological disease. The most common neurological syndrome associated with COVID-19 were encephalopathy (18.9%) and headache (13.7%). Pre-existing neurological disease and an elevated neutrophil count were the strongest predictors of developing any neurological complications. Death form COVID-19 was associated with age (OR 1.06, 95% CI 1.02–1.10, *P* = 0.001), invasive ventilation (OR 37.12, 95% CI 13.36–103.14), COVID-19-related-neurological complications (OR 3.24, 95% CI 1.28–8.21, *P* = 0.01), and elevated CRP level (OR 1.01, 95% CI 1.00–1.01, *P* = 0.01).

**Conclusions:** COVID-19 is associated with a wide range of neurological manifestations in people living in Saudi Arabia, with older individuals and those with underlying neurological disorders being most at risk. The presence of neurological complications was associated with increased mortality and poor outcomes.

## Introduction

The most common presentation of COVID-19 is that of a respiratory tract illness manifesting with fever, myalgia, cough, dyspnea, and fatigue (1). The disease severity ranges from asymptomatic or mild illness to severe respiratory failure requiring supportive care, intubation, and possibly death (1). The responsible pathogen is SARS-CoV-2, an RNA virus that enters the cells through ACE2 receptors (2). These receptors are found in the respiratory tract and other tissue such as the kidney, the gastrointestinal tract, vascular endothelial cells, and have been found in the central nervous system as well (3).

Neurological manifestations have increasingly become recognized in COVID-19 patients since the initial COVID-19 outbreak (4) and are rather common (5). Numerous case reports, in addition to cohort studies, have emerged since the start of the pandemic and revealed various common neurological manifestations such as headache, anosmia, encephalopathy, coma and stroke (6, 7). whereas Guillain-Barré Syndrome (GBS) (8), peripheral nerve and muscle disease (4), transverse myelitis (9) encephalitis and seizures were less commonly associated with COVID-19 disease (10–12).

The mechanism by which SARS-CoV-2 impairs neurological function is still not clearly understood. Neuropathological autopsy studies of patients with COVID-19 showed evidence of astrogliosis and inflammatory cell infiltration of the cerebellum and brainstem (13, 14). Autopsy studies provided little evidence of encephalitis or direct central nervous system damage caused by SARS-CoV-2 thus far (13). A recent autopsy study of 41 brains showed very low levels of virus in the brain parenchyma and determined that the pathological changes were likely from hypoxia and systemic inflammation (13). Moreover, histopathological studies revealed no signs of vasculitis (13, 14).

Experts have suggested other mechanisms that would more likely contribute to the development of the heterogeneous neurological manifestations associated with COVID-19 (15). For example, endothelial damage and coagulopathy are possible mechanisms involved in the increasing proportion of stroke incidence in COVID-19 patients (11, 15).

People from different ethnic backgrounds are experiencing different outcomes related to SARS-CoV-2 infection (16). For example, non-Hispanic black Americans are two times likely to die from COVID-19 than white Americans (16, 17). Several factors might contribute to this increased risk, such as lower socioeconomic status, the prevalence of chronic conditions, and possibly biological factors (18, 19). The prevalence of neurological complications associated with SARS-CoV-2 infection in Arab countries is still unknown. Our study aims to study the common neurological complications associated with COVID-19 in residents of Saudi Arabia and their relationship with clinical outcomes. We also examine which factors are associated with the development of neurological manifestations.

## Methods

### Participants

We reviewed all the COVID-19 cases admitted to King Saud University Medical City (KSUMC), a tertiary hospital in Riyadh, Saudi Arabia. We screened cases from March 2020 until the end of December 2020. We followed cases and controls until they were discharged from the hospital or died. The observation period ended in March 2021. We included cases if they were hospitalized with a positive RT-PCR for SARS-CoV-2 and presented with any of the following neurological syndromes or developed it during the incident hospital admission; (a) stroke or Transient Ischemic Attack (TIA), (b) encephalitis, (c) encephalopathy/delirium, (d) meningitis/meningism, (e) central nervous system vasculitis, (f) myelitis/myelopathy, (g) acute disseminated encephalomyelitis (ADEM), or (h) GBS or any other neurological complications. We included controls if they were admitted with COVID-19 illness with a positive RT-PCR for SARS-CoV-2 and did not present with or develop any of the above neurological complications during the observation period. We matched cases and controls based on basic demographics, and comorbidity profile. Study coordinators were trained on the definitions of the neurological syndromes and their manifestations. Case definitions are detailed in Appendix 1. A panel of three expert neurologists and a geriatrician reviewed and ascertained cases periodically. We excluded cases if there were disagreements between any panel members on fulfilling the criteria for the neurological syndromes of interest.

### Design

This is an observational prospective cohort study. We observed cases from the time of hospital admission until their discharge or death in the hospital.

### Ethics Approval

We collected all the data anonymously after receiving approval from King Saud University institutional review board with reference number IRB number 20/0539/IRB project number E-205076. Informed consent was not required based on our institutional review board's policies.

### Procedure

Data were collected and stored in a secure database with restricted access to the study authors. We used electronic medical records to collect basic demographics and other study variables. The primary outcomes of interest were death from any cause, survival, and survival with sequalae. The data that support the findings of this study are available from the corresponding author, upon reasonable request.

### Analysis

Continuous variables were reported as median and interquartile range (IQR), and categorical variables as numbers and proportions. The two groups of patients with and without COVID-19-related neurological complications were compared using Chi-square, Fisher's exact, or Mann-Whitney-U tests, as appropriate. Variables with a statistically significant *P*-value were entered in a multivariate logistic regression model as independent variables, whereas a COVID-19-related neurological complication (yes/no) was entered as a dependent variable. A separate multivariate logistic regression model was employed to assess variables independently associated with mortality. Collinearity between independent variables was assessed, and those with a variance inflation factor >5 were excluded. Odds ratios (ORs) and their 95% confidence intervals (CIs) were computed. Missing data were not imputed. A two-tailed *P*-value of < 0.05 was considered significant. Statistical analysis was conducted with the software SPSS (version 23, Chicago, IL).

## Results

In total, 497 patients (64% men) with COVID-19 were hospitalized from March 2020 to December 2020. The median (IQR) age was 53 (39–63) years, as shown in Table 1. The majority were Arabs (77.9%). The most common symptoms at presentation were cough (82.7%), fever (81.9%), and dyspnea (75.1), as shown in Table 2. The most commonly reported neurological complications were decreased level of consciousness (18.3%), confusion (15.5%), disorientation (15.7%), and headache (13.7%). The most common radiological finding was CXR infiltrates (bilateral 74.1%, unilateral 7.5%), Table 3.

**Table 1:** Characteristics of the hospitalized patients with the Novel Coronavirus (COVID-19) Infection.

**Variables**	**Median (IQR) or** ***N*** **(%)**			
	**All patients**	**Neurological complications**		
	**(*N* = 497)**	**No (*N* = 379)**	**Yes (*N* = 118)**	***P*-value**
**Demographics**				
Age, years	53 (39–63)	51 (37–61)	60 (48–71)	<0.001
Men	318 (64)	242 (63.9)	76 (64.4)	0.91
Women	179 (36)	137 (36.1)	42 (35.6)	
Body Mass Index (BMI)	28.7 (25.1–33.7)	28.8 (25.3–33.7)	28.3 (24.7–34.1)	0.96
Ethnicity (Arabs)	387 (77.9)	288 (78.3)	99 (88.4)	0.02
Chronic diseases	339 (68.2)	241 (64.6)	98 (83.1)	<0.001
Diabetes mellitus	220 (44.3)	154 (40.6)	66 (55.9)	<0.01
Hypertension	195 (39.2)	132 (34.8)	63 (53.4)	<0.001
Dyslipidemia	62 (12.5)	41 (10.8)	21 (17.8)	0.05
Connective tissue diseases	14 (2.8)	13 (3.4)	1 (0.8)	0.13
Pulmonary disease	50 (10.1)	36 (9.5)	14 (11.9)	0.46
Neurological disease	66 (13.3)	28 (7.4)	38 (32.2)	<0.001
Renal disease	25 (5)	16 (4.2)	9 (7.6)	0.14
Endocrinological disease	39 (7.8)	29 (7.7)	10 (8.5)	0.77
Gastrointestinal disease	17 (3.4)	14 (3.7)	3 (2.5)	0.77
Hepatic disease	4 (0.8)	2 (0.5)	2 (1.7)	0.24
Cardiac disease	76 (15.3)	49 (12.9)	27 (22.9)	0.01
Psychiatric disease	13 (2.6)	8 (2.1)	5 (4.2)	0.20
Hematological disease	17 (3.4)	12 (3.2)	5 (4.2)	0.57
Urological disease	7 (1.4)	3 (0.8)	4 (3.4)	0.06
Dermatological disease	5 (1.0)	1 (0.3)	4 (3.4)	0.01
Oncology disease	22 (4.4)	19 (5)	3 (2.5)	0.25
**Laboratory investigations**				
Hemoglobin gm/L (*n* = 496)	134 (118–144)	135 (119–145)	129 (109–142)	0.01
Neutrophils 10^9^/L (*n* = 484)	5.1 (3.2–7.4)	4.7 (3.1–7)	6.3 (3.6–9.6)	<0.001
Lymphocytes 10^9^/L (*n* = 486)	1.1 (0.7–1.5)	1.1 (0.8–1.5)	0.9 (0.6–1.4)	0.02
Platelets 10^9^/L (*n* = 495)	233 (178–299)	234 (180–297)	225 (172–313)	0.40
Creatinine mcmol/L (*n* = 495)	85 (65–111)	81 (61–102)	104 (81–174)	<0.001
Lactate Dehydrogenase (LDH) unit/L (*n* = 439)	397 (290–563)	387 (284–539)	430 (315–623)	0.01
Fibrinogen g/L (*n* = 261)	6.0 (4.9–7.1)	5.9 (4.6–7.1)	6.1 (5.2–7.4)	0.19
Procalcitonin ng/ml (*n* = 343)	0.13 (0.05–0.6)	0.11 (0.05–0.4)	0.23 (0.1–1.4)	<0.001
Troponin ng/ml (*n* = 360)	8.2 (2.1–23.9)	6.3 (1.5–16.5)	19 (7.3–41.8)	<0.001
Interleukin-6 (IL6) pg/ml (*n* = 257)	57 (22–139)	47 (19–127)	87 (46–192)	<0.01
D-Dimer, μg /mL (*n* = 453)	1.1 (0.64–2.02)	0.91 (0.59–1.6)	1.5 (0.9–3.6)	<0.001
Ferritin, μg/L (*n* = 453)	582.7 (192.4–1219.7)	589 (170–1206)	550 (218–1408)	0.38
C-Reactive Protein (CRP), mg/L (*n* = 430)	91 (41–142)	88 (40–136)	98 (42–174)	0.11
Erythrocyte Sedimentation Rate (ESR) mm/h (*n* = 297)	67 (43–93)	67 (45–93)	64 (38–96)	0.69
**Outcome**
Needed oxygen supplementation	368 (74.6)	269 (71.4)	99 (85.3)	<0.01
ICU admission	165 (33.3)	96 (25.3)	69 (59)	<0.001
Invasive ventilation	85 (17.1)	30 (7.9)	55 (47)	<0.001
Died	93 (18.7)	35 (9.2)	58 (49.2)	<0.001

**Table 2:** Clinical Presentation of hospitalized Patients with the Novel Coronavirus (COVID-19) Infection.

**COVID symptoms/signs**	***N* (%)**
Cough	411 (82.7)
Fever	407 (81.9)
Dyspnea	373 (75.1)
Diarrhea	120 (24.1)
Myalgia	119 (23.9)
Chest pain	99 (19.9)
Vomiting	98 (19.7)
Decreased level of consciousness	91 (18.3)
Disorientation	78 (15.7)
Confusion	77 (15.5)
Headache	68 (13.7)
Sore throat	72 (14.5)
Abdominal pain	59 (11.9)
Behavioral change	54 (10.9)
Anosmia	34 (6.8)
Ageusia	32 (6.4)
Seizure	12 (2.4)
Hallucinations	5 (1.0)
Abnormal movement	3 (0.6)
Visual symptoms	3 (0.6)
Photophobia	1 (0.2)
**Signs**	
Neck stiffness	2 (0.4)
Aphasia	5 (1.0)
Dysphagia	6 (1.2)
Dysarthria	8 (1.6)
Limb weakness	20 (4.0)
Sensory involvement	10 (2.0)

**Table 3:** Radiological investigations of hospitalized patients with the Novel Coronavirus (COVID-19) Infection.

**Radiological Investigations**	***N* (%)**
Chest X-ray (*n* = 464)	
Normal	85/464 (18.3)
Unilateral infiltrate	35/464 (7.5)
Bilateral infiltrate	344/464 (74.1)
Computed Tomography CT chest (*n* = 45)	
Normal	10/45 (22.2)
Unilateral infiltrate	4/45 (8.9)
Bilateral infiltrate	21/45 (46.7)
Unilateral ground glass opacity	2/45 (4.4)
Bilateral ground glass opacity	21/45 (46.7)
CT-PE Pulmonary embolism (*n* = 43)	
Negative	39 (90.7)
Positive	4 (9.3)
CT head (*n* = 74)	
Normal	44/74 (59.5)
Acute stroke	9/74 (12.2)
Intracranial hemorrhage (ICH)	3/74 (4.1)
Old infarct	16/74 (21.6)
Non-specific white matter hyperintensities	2/74 (2.7)
Magnetic resonance imaging MRI brain (*n* = 7)	
Normal	1/7
Acute stroke	1/7
ICH	1/7
Old infarct	3/7
Non-specific white matter hyperintensities	1/7
Cerebrospinal fluid CSF (*n* = 6)	
Increased white blood cell count	1 patient (cell count 10)
Increased Protein	2 patients (0.6 and 0.52)
Electromyography EMG (n = 3)	1 normal and 2 axonal sensory motor neuropathies

Neurological complications of COVID-19 were reported in 118 (23.7%) patients. Among all patients, there were 94 patients (18.9%) with encephalopathy and 16 patients (3.2%) with cerebrovascular accidents (CVA), as shown in Table 4. Patients with COVID-19-related neurological complications were older, more likely to have a chronic disease, particularly previous neurological disease, and required oxygen supplementation more frequently than those without neurological complications (Table 1).

**Table 4:** Complications in the hospitalized Patients with the Novel Coronavirus (COVID-19) Infection.

**Complications**	***N* (%)**
All complications	194 (39.0)
Neurological syndromes	118 (23.7)
A) Encephalopathy	94 (18.9)
B) Stroke	16 (3.2)
Large artery stroke	9 (1.8)
Small artery stroke	2 (0.4)
Unclassified	3 (0.6)
Transient Ischemic Attack (TIA)	2 (0.4)
C) Meningitis	2 (0.4)
D) Encephalitis	1 (0.2)
E) Central nervous system vasculitis	0 (0)
F) Myelitis/myelopathy	0 (0)
G) ADEM	0 (0)
H) GBS	0 (0)
Myopathy	4 (0.8)
Seizures	12 (2.4)
Brain herniation	5 (1.0)
Other unclassified neurological complications	5 (1.0)
Acute kidney injury	95 (19.1)
Sepsis/septic shock	78 (15.7)
Pulmonary embolism	21 (4.2)
Hepatic injury	16 (3.2)
Cytokine storm	12 (2.4)
Myocardial Infarction	11 (2.2)
Pneumothorax or emphysema	9 (1.8)
Thrombosis	8 (1.6)
Pleural effusion	2 (0.4)
Myopericarditis	1 (0.2)

Compared with patients without neurological complications, those with COVID-19-related neurological complications had significantly higher neutrophil count, higher D-dimer levels, serum creatinine, troponin, procalcitonin, LDH, and interleukin-6, and lower levels of hemoglobin and lymphocyte count (Table 1).

The regression model for the outcome “COVID-19-related neurological complications” included 251 patients, excluding those with missing data. The model explained 32.7 % of the variance in the dependent variable. The independent variables that showed significant association with COVID-19-related neurological complications were age (OR 1.03, 95% CI 1.009–1.058, *P* = 0.007), neutrophil count (OR 1.11, 95% CI 1.011–1.218, *P* = 0.029), and neurological disease at baseline (OR 6.6, 95% CI 2.74–16.10, *P* < 0.001), Table 5. The regression model for the outcome “mortality” included 238 patients, and the model explained 65.9 % of the variance in the dependent variables. The independent variables that demonstrated significant association with death were age (OR 1.06, 95% CI 1.02–1.10, *P* = 0.001), invasive ventilation (OR 37.12, 95% CI 13.36–103.14), COVID-19-related-neurological complications (OR 3.24, 95% CI 1.28–8.21, *P* = 0.01), and CRP (OR 1.01, 95% CI 1.00–1.01, *P* = 0.01), Table 6.

**Table 5:** Multivariate logistic regression analysis for the dependent variable COVID-19-related neurological complications (*n* = 251).

	**Neurological complications**			
			**95% CI**	
**Variable**	***P*-value**	**OR**	**Lower**	**Upper**
Age, years	0.01	1.03	1.01	1.06
Ethnicity (Arabs)	0.32	0.62	0.25	1.57
Neurological disease at baseline	<0.001	6.64	2.74	16.10
Cardiac disease at baseline	0.91	0.95	0.42	2.15
Needed Oxygen supplementation	1.00	1.00	0.29	3.42
Hemoglobin gm/L	0.82	1.00	0.98	1.02
Lymphocytes	0.29	1.21	0.85	1.73
Neutrophils	0.03	1.11	1.01	1.22
Creatinine mcmol/L	0.09	1.01	1.00	1.01
LDH_unit/L	0.49	1.00	1.00	1.01
Procalcitonin ng/ml	0.22	1.01	1.00	1.02
Troponin ng/ml	0.31	1.00	1.00	1.00
D-Dimer mcg/ml	0.30	1.04	0.97	1.12

**Table 6:** Multivariate logistic regression analysis for the dependent variable COVID-19-related death (*n* = 238).

	***P*-value**	**OR**	**95% C.I**	
			**Lower**	**Upper**
Age	0.001	1.06	1.02	1.10
Invasive Ventilation	<0.001	37.12	13.36	103.14
Overall COVID-19-related neurological complications	0.01	3.24	1.28	8.21
Neutrophil count 10^9^/L	0.43	0.96	0.86	1.07
CRP mgL	0.01	1.01	1.00	1.01
D Dimer mcg/ml	0.92	1.00	0.95	1.05
Fibrinogin g/L	0.99	1.00	0.90	1.11
Needed Oxygen supplementation	0.08	10.34	0.74	143.98

## Discussion

In our cohort of people living in Saudi Arabia who were hospitalized with COVID-19, almost a quarter of all patients had neurological complications. The most common neurological manifestation was encephalopathy, occurring in 19% of all hospitalized patients with COVID-19.

The strongest predictors of presenting with any neurological syndrome were having pre-existing neurological disease followed by elevated neutrophils count and advanced age. Having any neurological syndrome was significantly associated with the need for invasive ventilation and death. Whereas advanced age, mechanical ventilation elevated CRP, and the presence of a neurological complication were significantly associated with death.

Pre-existing neurological disease at baseline was strongly associated with in-hospital neurological complications in our cohort. Previous studies found that SARS-COV-2 infection can either exacerbate pre-existing neurological disorders or complicate COVID-19 disease (20, 21). Pre-existing neurological disorders, such as dementia, Parkinson disease, and stroke, are associated with a disrupted blood-brain barrier and vulnerability to infection and inflammatory states (22–24). SARS-COV-2 infection might reach the brain through olfactory nerves and other neurotropic or hematogenous routes (25). COVID-19 disease is associated with systemic inflammatory responses and cytokine production, which can reach the vulnerable brain and precipitate encephalopathy, as can the metabolic derangements resulting from organ failure or certain drugs (21, 25–28).

Encephalopathy/delirium was common in our cohort, notably in older adults who possibly have less cognitive reserve and thus more prone to develop delirium (29). The rate of encephalopathy in our cohort is similar to the rates reported by (5, 28). The pathobiology of encephalopathy in COVID-19 is interesting and not completely understood; however, the inflammatory response associated with COVID-19 disease can precipitate coagulopathy, and endotheliitis, which in turn can lead to downstream microvascular dysfunction manifesting with delirium and cognitive impairment (15, 30–32). Moreover, delirium in COVID-19 as in other settings could be multifactorial, triggered by multiple precipitating factors such as hypoxemia, change in environment, immobility, sedative agents, especially in older patients prone to delirium (25, 27, 33). It is not surprising to see this high frequency of encephalopathy in our cohort. A recent meta-analysis found that ~96% of COVID-19 patients had abnormal background activity on electroencephalography (26).

The frequency of meningitis and encephalitis were relatively low in our cohort with non-revealing CSF analysis or imaging studies to support that SARS-COV-2 is the etiological agent. In Lersy et al., CSF analysis showed modest White blood Cells (WBC) elevation in patients with encephalopathy similar to our cohort, but higher CSF WBC in patients with stroke (34). There is limited evidence to support the neurotropism of SARS-COV-2 (35–37). However, the systemic inflammatory response associated with severe COVID-19 disease described in our cohort, such as the high neutrophil count and other inflammatory markers, is likely the major contributor to the development of most the neurological complications seen here. Autopsy studies of brain tissues from COVID-19 infected patients revealed that neuroinflammatory changes are common findings among pathological studies. Additionally, evidence of microvascular dysfunction, coagulopathy, and hypoxia were also described (13, 14).

The prevalence of strokes in our cohort was not inconsistent with previous studies (4, 5, 12, 28, 38–40). Additionally, we found a similar distribution of stroke types as those reported previously by (41). Older patients in our cohort were more likely to develop vascular events as they were more likely to develop the other neurological complications. The association between age and neurological complications is not surprising and similar to that reported in the UK surveillance study (12).

Seizures occurred in our cohort within the range of previous reports (4, 42–44). Hypoxic or metabolic changes often trigger seizures (44). In one study, the most common EEG indication was altered mental status, and epileptiform discharges were more common in those with a history of epilepsy. Epileptiform discharges were even found in COVID-19 patients without pre-existing seizures (26).

We did not find an association between lymphopenia and poor outcomes or neurological complications, unlike most previous studies (4, 45, 46). On the other hand, neutrophilia was predictive of neurological complications in the multivariate logistic regression model. Neutrophils play a distinct role in cytokine production and restricting viral replication (47). The association between poor outcomes and high neutrophils count in our study is possibly related to the state of hyper inflammation induced by excessive release of cytokines, also known as “the cytokine storm” (45, 47, 48).

We found a lower number of anosmia, which possibly was infrequently reported by patients during the initial phase of the pandemic. Still, this finding is not inconsistent with previous literature, which reported a similar frequency of anosmia (39, 40, 49). Moreover, the frequency of the other general neurological symptoms such as headache, myalgia, and weakness were relatively low in our cohort but invariable to other reports of COVID-19 related neurological manifestations (4, 38, 50).

In our study, the group of patients with COVID-19 disease who did not have any neurological syndrome were significantly younger and had less chronic disease than those who had neurological complications. More importantly, those who did not present with any neurological complication during hospitalization were less likely to require invasive ventilation and more likely to survive from their COVID-19 disease. To our knowledge, this is the first study in Saudi Arabia to systematically and prospectively describe the predictors and outcomes of the common neurological complications associated with COVID-19 disease.

Our study has several limitations to consider. Although encephalopathy represented most of the neurological syndromes reported here, we did not use a validated tool to screen for in-hospital delirium. Therefore, we might have underreported COVID-19 patients presenting with hypoactive delirium, a very common form of delirium that usually presents with drowsiness and reduced level of consciousness rather than agitation and restlessness common in hyperactive delirium (51). Additionally, during the pandemic's initial phase, there was difficulty performing important diagnostic studies such as MRIs and lumbar punctures due to infection control measures, which possibly limited the accurate diagnosis of several neurological complications.

## Conclusion

In conclusion, COVID-19 is associated with many different neurological manifestations in our population of Saudi Arabian residents. The rates of neurological complications are similar to previous reports in other regions, with older individuals and those with underlying neurological disorders being most at risk. Neurological complications are associated with increased mortality and worse outcomes in patients with COVID-19 disease. Therefore, special attention needs to be given to patients who already have underlying neurological disorders at the time of acquiring COVID-19 infection, to screen for the common neurological complications associated with COVID-19 during the early hospitalization phase and implement the preventive measures known to improve outcomes for the neurological complications.

## Data Availability Statement

The raw data supporting the conclusions of this article will be made available by the authors, without undue reservation.

## Ethics Statement

The studies involving human participants were reviewed and approved by Ethical approval was received approval from King Saud University institutional review board with reference number IRB number 20/0539/IRB project number E-205076. Written informed consent for participation was not required for this study in accordance with the national legislation and the institutional requirements.

## Author Contributions

WA, MA, and TM: conceived and designed the study. AAlq, NA, and WA: collected the data. MA and WA: performed the data analysis. WA, TM, MA, and AAlh: wrote the manuscript. All authors contributed to the article and approved the submitted version.

## Funding

The authors extend their appreciation to College of Medicine Research Center, Deanship of Scientific Research at King Saud University for funding this research work.

## Conflict of Interest

The authors declare that the research was conducted in the absence of any commercial or financial relationships that could be construed as a potential conflict of interest.

## Publisher's Note

All claims expressed in this article are solely those of the authors and do not necessarily represent those of their affiliated organizations, or those of the publisher, the editors and the reviewers. Any product that may be evaluated in this article, or claim that may be made by its manufacturer, is not guaranteed or endorsed by the publisher.

## Abbreviations

COVID-19: coronavirus disease 2019
GBS: guillain-barré syndrome
CNS: central nervous system
TIA: transient ischemic attack
CVA: cerebrovascular accidents
ADEM: acute disseminated encephalomyelitis
CRP: C-reactive protein
IL6: interleukin-6.
