# Supplementary material for: The Common Neurological Presentations and Clinical Outcomes of Coronavirus Disease 2019 in Saudi Arabia
Source: Front Neurol. 2021 Sep 8;12:737328. doi: 10.3389/fneur.2021.737328 (PMC8455892; doi:10.3389/fneur.2021.737328)
Supplement: Supplementary file 1 [file Data_Sheet_1.docx]

**Appendix 1**

**Table 1. WHO COVID-19 Case definitions**

|  | **Confirmed** | | | | **Probable** | | **Suspected** | |  |
| --- | --- | --- | --- | --- | --- | --- | --- | --- | --- |
| **WHO COVID-19 case definitions, taken from World Health Organization. COVID-19: situation report, 95.1** | **A person with laboratory confirmation2 of SARS-CoV-2 infection, irrespective of clinical signs and symptoms.** | | | | **A suspect case, for whom testing for the COVID-19 virus is inconclusive** | | **A patient with acute respiratory illness (fever and at least one sign/symptom of respiratory distress) AND history of travel to or residence in a location reporting community transmission of COVID-19 disease during the 14 days prior to onset** | |  |
|  |  | | | | **OR** | | **OR** | |  |
|  | **Confirmatory tests include a nucleic acid amplication test (e.g. RT-PCR) or validated antibody test,** | | | | **A suspect case, for whom testing could not be performed for any reason** | | **A patient with acute respiratory illness (fever and at least one sign/symptom of respiratory distress) AND having been in contact with a confirmed or probable case in the last 14 days prior to symptom onset** | |  |
|  |  | | | |  | | **OR** | |  |
|  | **·       In an area WITH established circulation of virus:  one positive RT-PCR test or identification of virus on sequencing. One or more negative tests do not rule out infection if clinical suspicion.** | | | |  | | **A patient with severe acute respiratory illness (fever and at least one sign/symptom of respiratory distress AND requiring hospitalisation) AND in the absence of an alternative explanation that fully explains the clinical presentation** | |  |
|  | **·       In an area WITHOUT established circulation of virus: one positive RT-PCR test for two different viral genome targets, OR one positive result with partial or whole genome sequencing** | | | |  | |  | |  |
|  | |  |  |  | |  | |  | |
|  | | **Table 1. WHO COVID-19 Case definitons** | | | | | |  | |

Table 2. **Provisional case definitions for neurological diseases associated with SARS-CoV-2 infection, based on previously established principles ^3,4,5^**

|  | | | **Confirmed** | **Probable** | | **Possible** | | |
| --- | --- | --- | --- | --- | --- | --- | --- | --- |
| **SARS-CoV-2 meningitis, encephalitis, myelitis/myelopathy**** | | | SARS-CoV-2 detected in CSF/ brain tissue †, | SARS-CoV-2 detected in respiratory or other non-CNS sample ‡, | | Patient meets suspected case definition of COVID-19 according to national or WHO guidance (as below), based on clinical symptoms and epidemiological risk factors. | | |
|  |  |  | OR | OR | |  | | |
|  |  |  | Evidence of SARS-CoV-2-specific intrathecal antibody; | Evidence of SARS-CoV-2-specific antibody in serum indicating acute infection+§; | | In the context of known community SARS-CoV-2 transmission, supportive features* include: | | |
|  |  |  |  |  | | *Clinical:* new onset of least one of: cough, fever, muscle aches, loss of smell, loss of taste; | | |
|  |  |  | AND | AND | | *Laboratory*: lymphopenia, raised d-dimer; | | |
|  |  |  | No other explanatory pathogen or cause found | No other explanatory pathogen or cause found | | *Radiological*: evidence of unilateral or bilateral abnormalities consistent with infection or inflammation (e.g. ground glass changes) | | |
| ***These supportive features are suggestions based on published information to date (24/04/2020); they are likely to need refining as more data emerge.** | | | | | | | | |
|  | | |  |  | |  | | |
|  | | | **Strong association** | **Probable association** | | **Possible association** | | |
| **Acute disseminated encephalomyelitis** (ADEM) associated with SARS-CoV-2 infection** | | | N/A | Neurological disease onset <= 6 weeks after acute infection, | | Neurological disease onset <= 6 weeks after acute infection, | | |
|  |  |  |  | AND | | AND | | |
|  |  |  |  |  |  |  |  |  |
|  |  |  |  | SARS-CoV-2 RNA detected in any sample, | | SARS-CoV-2 RNA detected in any sample; | | |
|  |  |  |  | OR | | OR | | |
|  |  |  |  | Antibody evidence of acute SARS-CoV-2 infection; | | Antibody evidence of acute SARS-CoV-2 infection; | | |
|  |  |  |  | AND | | AND | | |
|  |  |  |  | No evidence of other commonly associated causes | | Evidence of other commonly associated causes | | |
| **Guillain-Barré syndrome** and other acute neuropathies associated with SARS-CoV-2 infection** | | | N/A | Neurological disease onset <= 6 weeks after acute infection, | | Neurological disease onset <= 6 weeks after acute infection, | | |
|  | |  |  | **AND** | | **AND** | | |
|  |  |  |  |  |  |  |  |  |
|  |  |  |  | SARS-CoV-2 RNA detected in any sample; | | SARS-CoV-2 RNA detected in any sample; | | |
|  |  |  |  | **OR** | | **OR** | | |
|  |  |  |  | Antibody evidence of acute SARS-CoV-2 infection; | | Antibody evidence of acute SARS-CoV-2 infection; | | |
|  |  |  |  |  |  |  |  |  |
|  |  |  |  | **AND** | | **AND** | | |
|  |  |  |  | No evidence of other commonly associated causes ¶ | | Evidence of other commonly associated causes ¶ | | |
| **Stroke or CNS vasculitis** associated with SARS-CoV-2 infection** | | | *CNS Vasculitis:* | *All other stroke types:* | | *CNS Vasculitis:* | | |
|  | | | SARS-CoV-2 detected in CSF/brain tissue†; | SARS-CoV-2 detected in CSF or other sample‡; | | SARS-CoV-2 detected in CSF/brain tissue†; | | |
|  |  |  | **OR** | **OR** | | **OR** | | |
|  |  |  | Evidence of SARS-CoV-2-specific intrathecal antibody; | Evidence of SARS-CoV-2-specific antibody in serum indicating acute infection; | | Evidence of SARS-CoV-2-specific intrathecal antibody; | | |
|  |  |  |  |  |  |  | | |
|  |  |  | **AND** | **AND** | | **AND** | | |
|  |  |  |  |  |  |  | | |
|  |  |  | The presence of histopathological features of angiitis within the brain | No other known traditional cardiovascular risk factors¥ | | Laboratory and imaging support for brain inflammation (MR scan evidence compatible with CNS vasculitis with characteristic angiographic changes; elevated levels of cerebrospinal fluid protein and/or cells, and/or the presence of oligoclonal bands) | | |
|  |  |  |  |  |  |  | | |
|  |  |  | **AND** |  |  | **AND** | | |
|  |  |  | No other explanatory pathogen or cause found |  |  | No other explanatory pathogen or cause found | | |
|  |  |  |  |  |  |  | | |
|  |  |  |  |  |  | *All other stroke types:* |  |  |
|  |  |  |  |  |  | SARS-CoV-2 detected in CSF or other sample; | | |
|  |  |  |  |  |  | **OR** | | |
|  |  |  |  |  |  | Evidence of SARS-CoV-2-specific antibody indicating acute infection; | | |
|  |  |  |  |  |  |  |  |  |
|  |  |  |  |  |  | **AND** |  |  |
|  |  |  |  |  |  |  |  |  |
|  |  |  |  |  |  | Other traditional cardiovascular risk factors¥ | | |
|  |  | | | | | |  | |
|  | † detection in CSF or brain tissue by PCR, culture, or immunohistochemistry, as appropriate; ‡ detection in non-CNS sample by PCR or culture. § Serological evidence of acute infection can be defined as i) detection of IgM, or ii) IgG seroconversion or iii) >=4-fold rise in antibody titres in paired acute and convalescent serum samples. ¶ These include: infection with one of *Campylobacter jejuni, Mycoplasma pneumoniae,  Cytomegalovirus* (CMV), Epstein–Barr virus (EBV), hepatitis E virus, Zika virus, or HIV; or vaccination in the last 6 weeks. Associated causes may differ depending on geographical location. ¥ traditional cardiovascular risk factors include; hypertension, current smoker, diabetes, hypercholesterolemia, and atrial fibrillation. | | | | | |  |  |
|  | **Table 2. Provisional case definitions for neurological diseases associated with SARS-CoV-2 infection, based on previously established principles ^3,4,5^** | | | | | | |  |

**References:**

1. World Health Organization. COVID-19: situation report, 95. Link: <https://www.who.int/docs/default-source/coronaviruse/situation-reports/20200424-sitrep-95-covid-19.pdf?sfvrsn=e8065831_>4

2. WHO. World Health Organization. Laboratory testing for coronavirus disease 2019 (COVID-19) in suspected human cases. Interim Guide 2020., Link: https://www.who.int/emergencies/diseases/novel-coronavirus-2019/technical-guidance/laboratory-guidance

3. Granerod J, Cunningham R, Zuckerman M, et al. Causality in acute encephalitis: defining aetiologies. Epidemiol Infect 2010. DOI:10.1017/s0950268810000725.

4. Solomon T, Dung NM, Vaughn DW, et al. Neurological manifestations of dengue infection. Lancet 2000; 355: 1053–9.

5. Mehta R, Soares CN, Medialdea-Carrera R, et al. The spectrum of neurological disease associated with Zika and chikungunya viruses in adults in Rio de Janeiro, Brazil: A case series. PLoS Negl Trop Dis 2018; 12: e0006212.
